# Percutaneous stone removal using cobra-shaped sheath and cholangioscopy for multiple hepatolithiasis with choledochoduodenal anastomotic stenosis

**DOI:** 10.1016/j.radcr.2022.03.007

**Published:** 2022-04-04

**Authors:** Fumio Chikamori, Shigeto Shimizu, Satoshi Ito, Michiyo Okazaki, Nobuyuki Tanida, Niranjan Sharma

**Affiliations:** a Department of Surgery, Japanese Red Cross Kochi Hospital, 1-4-63-11 Hadaminamimachi, Kochi, 780-8562, Japan; b Department of Radiology, Japanese Red Cross Kochi Hospital, 1-4-63-11 Hadaminamimachi, Kochi, 780-8562, Japan; c Department of Gastroenterology, Japanese Red Cross Kochi Hospital, 1-4-63-11 Hadaminamimachi, Kochi, 780-8562, Japan; d Adv Train Gastroint & Organ Transp Surgery, 12 Scotland Street Dunedin, 9016, New Zealand

**Keywords:** Cobra-shaped sheath, Percutaneous stone removal, Hepatolithiasis, Choledochoduodenostomy, choledochoenterostomy, Percutaneous balloon dilatation

## Abstract

Management of multiple hepatolithiasis with choledochoenteral anastomotic stenosis remains difficult and time-consuming. We report a case of a 77-year-old man with severe right hypochondoralgia, treated with percutaneous transhepatic balloon dilatation of choledocoduodenal anastomotic stenosis and percutaneous stone removal using 8Fr. cobra-shaped sheath and cholangioscopy. Hilar hepatic stones were pushed out into the duodenum through the dilated anastomosis using 5Fr. balloon catheter covered with the sheath and cholangioscopy. For stones located in the left, right anterior and aberrant right posterior hepatic ducts, a guidewire and a removal balloon catheter were inserted by using the cobra-shaped sheath. Stones pulled from the intrahepatic bile ducts to the common hepatic duct were pushed out into the duodenum. Clearance of intrahepatic bile duct stones was confirmed by balloon-occluded cholangiography using the cobra-shaped sheath and 6Fr. balloon catheter. The use of cobra-shaped sheath improved percutaneous stone removal, but the procedure needs further improvement.

## Introduction

Hepatolithiasis is a serious complication observed in a long-term follow-up after choledochoenterostomy such as choledochojejunostomy or choledochoduodenostomy [1,2]. There are some treatments for hepatolithiasis [3], [4], [5], [6], however, the ideal treatment for multiple hepatolithiasis with anastomotic stenosis after choledochoenterostomy has not been established. We report a case of multiple hepatolithiasis secondary to choledochoduodenal anastomotic stenosis that was treated with less invasive percutaneous stone removal using cobra-shaped sheath and cholangioscopy.

### Case report

A 77-year-old man was referred to our department with severe right hypochondoralgia. He had surgery for early gastric cancer at the age of 69.

At that time, he suffered a bile duct injury; and underwent cholecystectomy, choledochoduodenostomy, distal gastrectomy, and Roux-en-Y gastrojejunostomy. Post-surgery, he often suffered from recurrent cholangitis. During his latest recurrence of cholangitis with jaundice and leukocytosis, he was admitted to a nearby hospital and was treated with antibiotics. He was on long-term diuretics for chronic heart failure.

On admission, he was markedly emaciated, his temperature was 39.6°C and his blood pressure was 127/78 mmHg. His height was 165 cm, body weight was 38.4kg, and body mass index was 14.1 kg/m^2^. He had right upper-quadrant abdominal tenderness. Laboratory results showed a hemoglobin of 10.7 g/dL (normal range, 13.5-17.4); a white blood cell count(WBC) of 6230/μL (3500-8000); a platelet count of 17.2 × 10^4^/μL (12.3-33.1); a total bilirubin of 1.1 mg/dL (0.3-1.3); an alanine transaminase (ALT) of 31 IU/L (10-32); an aspartate aminotransferase level (AST) of 20 IU/L (5-27); lactate dehydrogenase (LDH) of 139U/L (106-211); alkaline phosphatase (ALP) of 579 U/L (38-113); γ-glutamyl transpeptidase (γ-GTP) of 489 U/L (11-64); blood urea nitrogen (BUN) of 20.6 mg/dL (8–20); creatinine of 0.80 mg/dL (0.36–1.06); prothrombin time (PT) of 12.8 sec (9-12); PT% of 86.8% (70-130); international normalized ratio (INR) of 1.1; activated partial thromboplastin time (APTT) of 28.7sec (20-35); procalcitonin (PCT) of 1.44 ng/mL (0-0.49); C-reactive protein (CRP) of 2.45 mg/dL (<0.16); brain natriuretic peptide (BNP) of 418.5 ng/mL (0-18.4). Based on the clinical findings and laboratory work up, recurrent cholangitis with chronic heart failure and anemia was suspected. Antibiotics were given as initial treatment

**Fig. 1:**
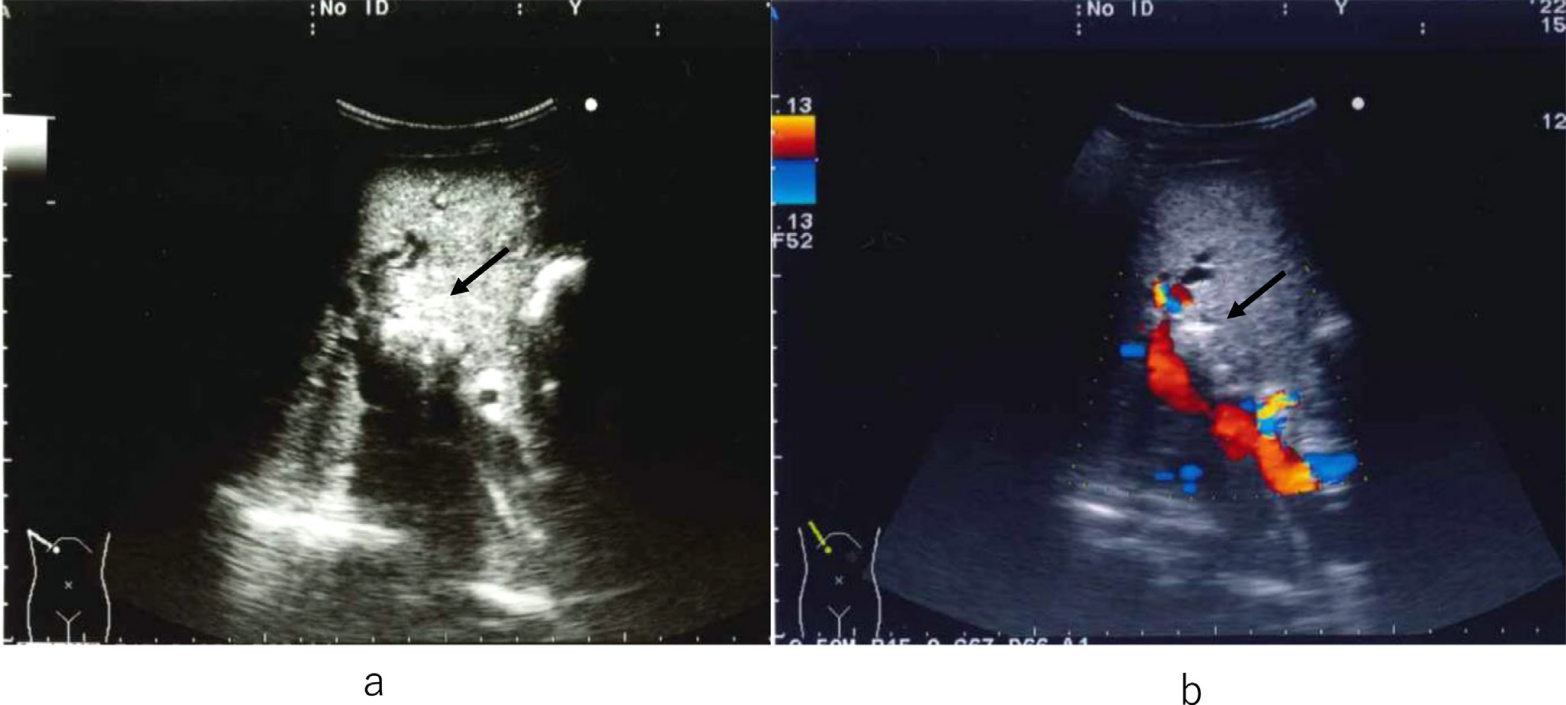
(A) Abdominal US shows high echoic lesions with acoustic shadow (arrow) (B) Color Doppler US before PTBD shows high echoic lesions (arrow) on the ventral side of the portal vein.

**Fig. 2:**
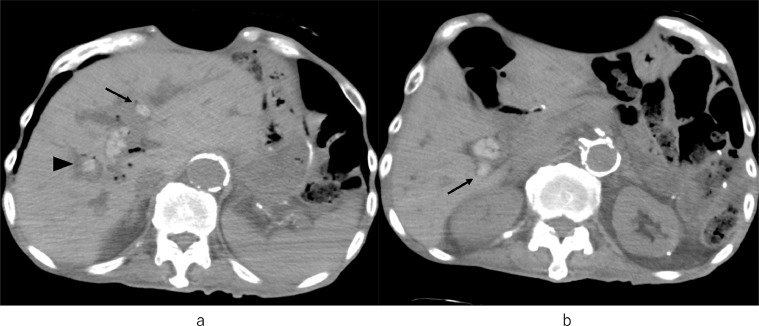
(A) Abdominal plain CT shows multiple stones in the left (arrow) and right anterior hepatic ducts (arrowhead) with biliary dilatation. (B) Abdominal plain CT shows multiple stones in the common hepatic duct and aberrant right posterior hepatic duct (arrow).

Abdominal ultrasonography (US) showed diffuse hyperechoic lesions in the liver and biliary dilatation (Fig. 1A). Plain computed tomography (CT) showed multiple stones in the left, right anterior, and aberrant right posterior hepatic ducts with biliary dilatation (Fig. 2A,B). The abdominal magnetic resonance imaging (MRI) and magnetic resonance cholangiopancreatography (MRCP) showed multiple hepatolithiasis in the bilateral intrahepatic bile ducts.

**Fig. 3:**
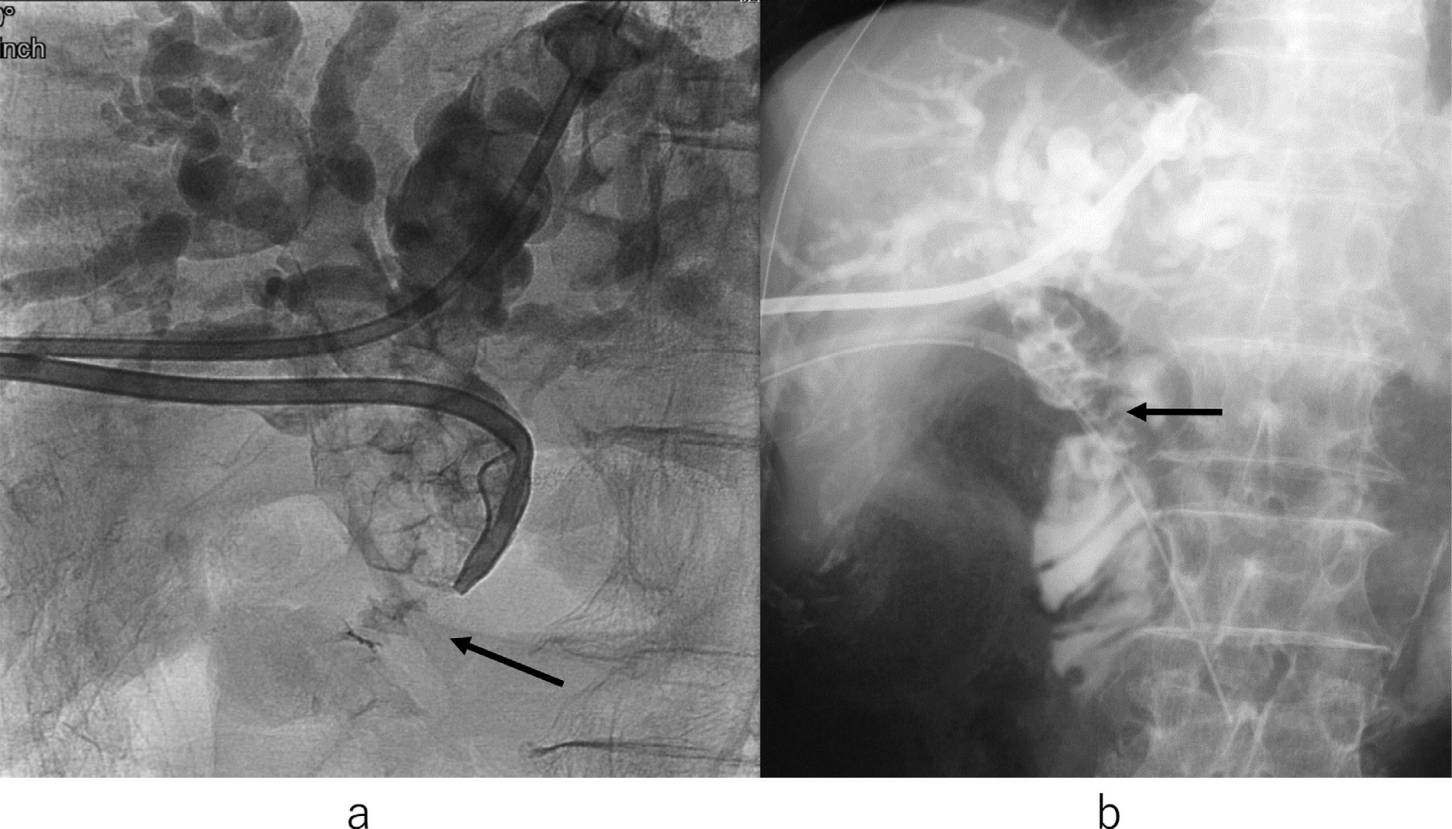
(A) Cholangiogram shows multiple hepatolithiasis and choledochoduodenal anastomotic stenosis (arrow). (B) Cholangiogram shows internal-external drainage tube passed through the choledochoduodenal anastomotic stenosis (arrow).

Percutaneous transhepatic biliary drainage (PTBD) was planned on the 4th day of hospitalization. Before PTBD, color Doppler US was performed to avoid vessel puncture (Fig. 1B). Percutaneous transhepatic cholangiography revealed multiple hepatolithiasis and choledocoduodenal anastomotic stenosis. Bile duct stones were distributed bilaterally in multiple segments. After introducing a guidewire into the bile duct through the puncture needle, first PTBD 8Fr. tube was inserted into the distal side of the left hepatic duct. Second PTBD 10Fr. tube was inserted into the common hepatic duct (Fig. 3A). The first tube was expected to decompress the bile duct and the second tube was expected to serve as a stone removal. The culture of bile showed the presence of Escherichia coli, Aeromonas caviae, and Enterococcus faecium.

On the 11th day, a guidewire from the second PTBD tube could not be passed through the choledochoduodenal anastomotic stenosis percutaneously. On the 19th day, a guidewire from the second PTBD tube could be passed through the stenosis by single-balloon endoscopy assistance. The second PTBD tube was replaced with 14Fr. internal-external biliary tube along the guidewire. On the 26th day, the tube was replaced with 18Fr. internal-external biliary tube (Fig. 3B).

**Fig. 4:**
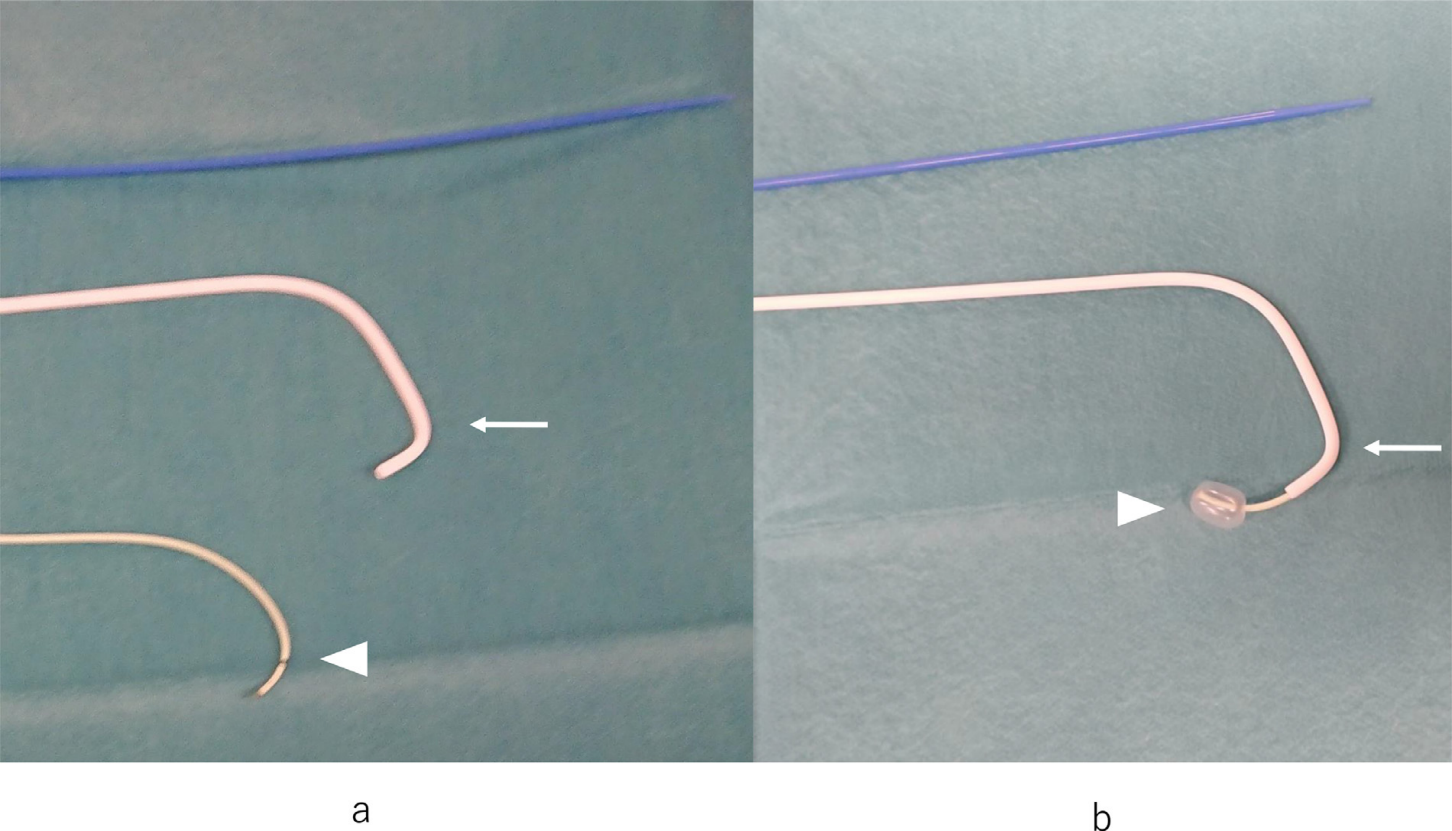
(A) 8Fr. cobra-shaped sheath (arrow) and 5Fr.balloon catheter (arrowhead). (B) Inflated balloon catheter (arrowhead) through the sheath with a natural U-turn shape (arrow).

**Fig. 5:**
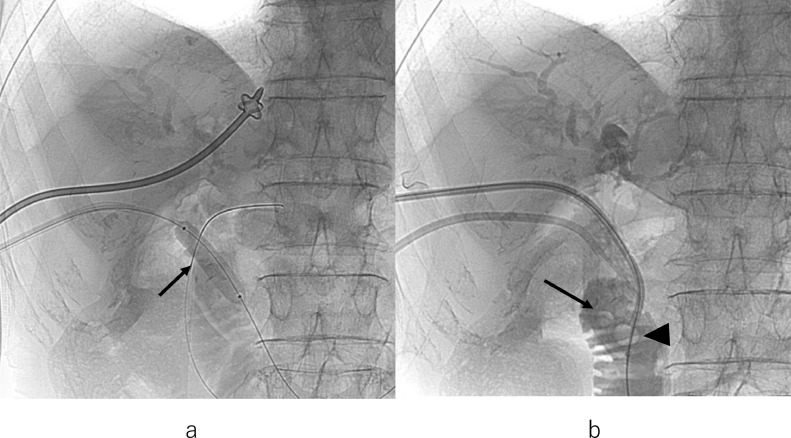
(A) Balloon dilation of the stenotic choledochoduodenal anastomosis (arrow). (B) Cholangiogram shows stones pushed out into the duodenum (arrow) using a removal balloon covered with the sheath (arrowhead).

**Fig. 6:**
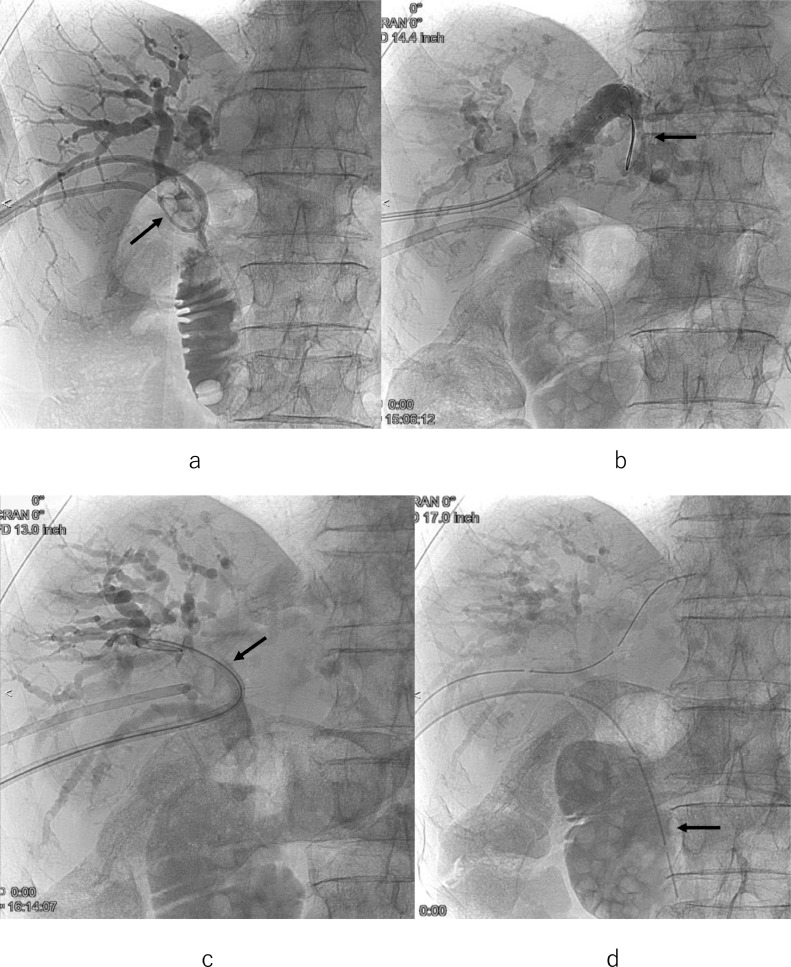
(A) Removal balloon catheter inserted by utilizing the cobra-shaped sheath for stones located in the aberrant right posterior hepatic duct (arrow). (B) Removal balloon catheter introduced along the guidewire, crossing the stones into the peripheral left hepatic duct (arrow). (C) Guidewire and removal balloon catheter inserted by utilizing the cobra-shaped sheath for stones located in the right anterior hepatic duct (arrow). (D) Cholangiogram after the procedure shows multiple stones in the duodenum (arrow).

On the 32nd day, percutaneous stone removal procedures were performed using an 8Fr. cobra-shaped sheath (Fig. 4A,B) [7], [8], [9]. The 8Fr. cobra-shaped sheath was inserted into the common hepatic duct. The stenotic choledochoduodenal anastomosis was dilated with a balloon 10mm in diameter for 10 minutes (Fig. 5A). Then hilar hepatic stones were pushed out into the duodenum through the dilated anastomosis using 5Fr. balloon catheter covered with the sheath and cholangioscopy (Fig. 5B). After that, by using the sheath via the 1st PTBD route, a removal balloon catheter was introduced along the guidewire, crossing the stones into the peripheral right posterior and left bile ducts (Fig. 6A,B). The balloon was inflated and stones were pulled into the common hepatic duct. For stones located in the right anterior branches, a guidewire and a removal balloon catheter were inserted by using the cobra-shaped sheath via the 2nd PTBD route (Fig. 6C). Then, the balloon was inflated and stones were pulled into the common hepatic duct again. Stones moved from the intrahepatic bile duct to the common hepatic duct were pushed out into the duodenum in the same way as described above (Fig. 6D). Infrared spectroscopy revealed that the bile duct stone component was 61% bilirubin calcium and 39% fatty acid calcium.

**Fig. 7:**
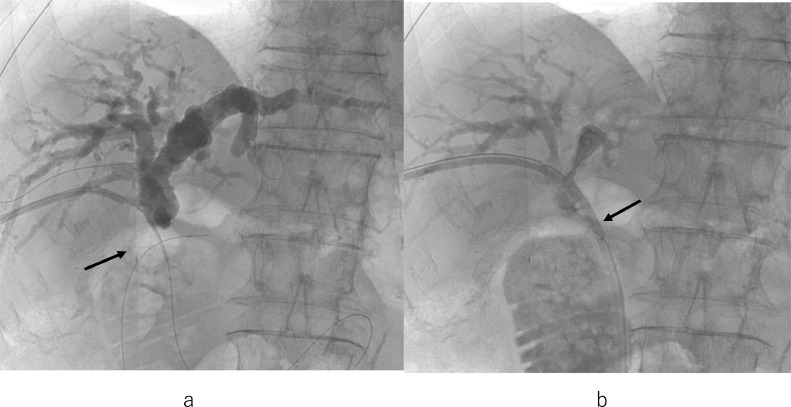
(A) Balloon-occluded cholangiogram using cobra-shaped sheath and 6Fr. balloon catheter shows clearance of intrahepatic bile duct stones. The contrast medium is injected through the sheath. An inflated balloon 2cm in diameter to occlude dilated choledochoduodenal anastomosis is indicated by an arrow. (B) Cholangiogram shows good bile flow through the trans-anastomotic 18Fr. internal-external biliary drainage tube (arrow).

On the 47th day, for residual stones stuck in the intrahepatic bile ducts, percutaneous transhepatic cholangioscopic lithotripsy (PTCSL) using electrohydraulic lithotripsy (EHL) [3,4] was performed. Clearance of intrahepatic bile duct stones was confirmed by balloon-occluded cholangiography using the cobra-shaped sheath and 6Fr. balloon catheter (Fig. 7A). Trans-anastomotic 18Fr. internal-external biliary drainage tube via the 1st PTBD route was left in situ to ensure bile flow through the dilated choledochoduodenal anastomosis (Fig. 7B). The indwelling tube will be left at the site for more than 3 months. Another tube via the 2nd PTBD route was removed. ALP, an indicator of cholestasis, normalized to 75 U/L after treatment. He was discharged on the 55th day.

## Discussion

We reported a case of multiple hepatolithiasis that developed 7 years after choledochoduodenostomy for bile duct injury. The patient was treated with percutaneous transhepatic balloon dilatation of choledocoduodenal anastomotic stenosis and percutaneous stone removal using cobra-shaped sheath and cholangioscopy.

Hepatolithiasis is defined as the presence of stones in the bile duct peripheral to the confluence of the right and left hepatic ducts. Its prognosis is poor due to complications including recurrent intrahepatic stones, recurrent cholangitis, biliary cirrhosis, liver atrophy, and intrahepatic cholangiocarcinoma [10], [11], [12]. Intrahepatic bile duct stones formation is the long-term serious complication of choledochoenterostomy such as choledochojejunostomy or choledochodudenostomy [1,2]. In this case, biliary stasis secondary to choledochoduodenal anastomotic stenosis was considered to be the cause of biliary infection, recurrent cholangitis, and stone formation. The management of multiple hepatolithiasis with choledochoenteral anastomotic stenosis remains a challenging task. There are some treatments such as revision of the choledocoenterostomy [13], PTCSL using EHL [3,4], and hepatectomy [14,15], however, multiple hepatolithiasis after choledochoenterostomy is still difficult to treat. No matter which treatment we choose, the stone removal procedure is not easy, it takes a long time, and it is surgically invasive. Therefore, it is important to devise more effective and less invasive treatment techniques.

Cobra-shaped sheath was used for stone removal, and it was useful for guiding the stone removal catheter to the periphery of most of the liver segments. The 8 Fr. cobra-shaped sheath was developed for the treatment of gastric varices [7]. Its use in percutaneous papillary balloon dilatation for cholecytocholedocholithiasis has been reported [8,9]. The sheath contributes to ensuring the operability and strength of the balloon catheter in stone removal. We adopted this sheath for multiple hepatolithiasis with choledochoduodenal anastomotic stenosis.

Some patients with hepatolithiasis cannot tolerate surgery due to the severity of the condition. It cannot be treated endoscopically because of previous gastrointestinal surgery. Our patient had chronic heart failure and previous surgery. Percutaneous radiological stone removal is an option in a small number of patients in whom endoscopic techniques are unsuccessful or impossible. A major advantage of the percutaneous approach compared to the endoscopic approach is the ease of repeated stone removal. Also, PTCSL using EHL in combination with percutaneous radiological stone removal was particularly useful for residual stones.

Monitoring of choledochoduodenal anastomosis is important during follow-up after stone removal. An internal-external drainage tube was left to monitor anastomosis and to prevent cholangitis and anastomotic restenosis.

The use of the cobra-shaped sheath improved the percutaneous stone removal, but the procedure needs further improvement.
